# Training Programs for Dementia Care Staff and Aggression in Nursing Homes: Secondary Analysis of a Cross-Sectional Online Survey

**DOI:** 10.2196/94077

**Published:** 2026-07-10

**Authors:** Reed Bratches, Tamara Backhouse, Shaoqing Ge, Megan Hebdon, Dalton Riegel, Noah Stein, Katie Trainum, Rita Jablonski

**Affiliations:** 1 School of Nursing, University of Alabama at Birmingham, 1720 University Boulevard, Birmingham, AL, 35294, United States, 1 205-975-8729; 2 School of Health Sciences, University of East Anglia, Norwich, England, United Kingdom; 3 The University of Texas at Austin, Austin, TX, United States; 4 University of Utah, Salt Lake City, UT, United States; 5 Franklin & Marshall College, Lancaster, PA, United States; 6 Center for healthcare Evaluation, Research, and Promotion, Corporal Michael J. Crescenz VA Medical Center, University of Pennsylvania, Philadelphia, PA, United States

**Keywords:** dementia care workforce, nursing home, assisted living, behavioral symptoms of dementia, care-resistant behavior

## Abstract

**Background:**

Training programs help dementia care staff handle residents’ behavioral symptoms in nursing homes and assisted living facilities. However, it is unclear how feeling well-prepared from such training relates to experiences of physical and verbal aggression from residents.

**Objective:**

This study aimed to determine whether perceived training preparedness was related to experiences of physical and verbal aggression from residents in nursing homes and assisted living facilities using data from a large, nationally representative dementia care workforce sample.

**Methods:**

This study was an analysis of the 2024 National Dementia Workforce Study, a cross-sectional online survey of the national dementia care workforce. Weighted regression modeling was used to identify associations between feeling well-prepared by training programs and experiencing physical or verbal aggression.

**Results:**

Dementia care staff in nursing homes (weighted n=896,320) commonly reported experiencing physical (365,452/896,320, 40.8%) and verbal (470,536/896,320, 52.5%) aggression from residents with dementia, whereas more than two-thirds (614,561/896,320, 68.6%) reported feeling that their training prepared them well to manage resident behaviors. Staff in assisted living facilities (n=738,856) less commonly reported experiencing physical (198,964/738,856, 26.9%) and verbal (223,343/738,856, 30.2%) aggression, whereas almost three-quarters (527,621/738,856, 71.4%) reported feeling that their training prepared them well to manage resident behaviors. In regression modeling in nursing homes, feeling well-prepared by training for managing resident behaviors was associated with a lower likelihood of experiencing physical aggression (odds ratio [OR] 0.4, 95% CI 0.223-0.711; *P*=.01) and verbal aggression (OR 0.5, 95% CI 0.299-0.844; *P*=.02). In assisted living facilities, feeling well-prepared by training for managing resident behaviors was associated with a lower likelihood of experiencing physical (OR 0.14, 95% CI 0.04-0.55; *P*=.01) and verbal (OR 0.07, 95% CI 0.0134-0.354; *P*=.001) aggression.

**Conclusions:**

While physical and verbal aggression from residents with dementia is common in nursing homes and assisted living facilities, nationally representative weighted regression modeling highlights an association between feeling well-prepared to handle resident behaviors and a lower likelihood of experiencing aggression. As the data used were derived from a cross-sectional survey, the causal link between these 2 factors should be explored in interventional studies. Our results indicate that effective training programs are associated with worker-reported reduced experiences of physical and verbal aggression in both nursing homes and assisted living facilities. The specific components of training programs that may support effectiveness remain unknown.

## Introduction

Aggression is a behavioral symptom of dementia that manifests as physically or verbally hostile or violent behavior [1]. Physical aggression includes striking, kicking, hair pulling, spitting, and throwing things, whereas verbal aggression includes swearing, insulting, threatening, or verbal sexual harassment [2]. Physically and verbally aggressive behaviors are common in residential facilities such as nursing homes and assisted living facilities, with estimates indicating aggression in 51% of patients with dementia in these facilities [3]. Staff exposure to aggression is associated with a higher likelihood of burnout and physical harm, including bruising and lacerations, and contributes to workforce turnover [4].

General nursing training programs rarely include focused training on managing the behavioral symptoms of dementia, including aggression [5]. While some licensed professional roles (eg, registered nurses and licensed practical nurses) require prerequisites and standardized competencies, the unlicensed direct care workforce receives limited formal education and highly variable on-the-job training. Studies and workforce reviews describe unlicensed direct care workers as underprepared for dementia-related agitation and aggression despite providing hands-on care to people with dementia [6,7]. In nursing education, dementia-specific content is inconsistently integrated into prelicensure curricula and taught under broad geriatric instruction rather than as a distinct competency, although proposed dementia-specific training programs have identified the management of aggression and agitation as a core competency for future training development [8].

There is a gap in the evidence on the effectiveness of training on the experience of physical or verbal aggression in nursing home or assisted living facility staff. Demonstrated differences in the effect of training programs are unclear, and studies are limited by small, nongeneralizable samples [9]. There is an opportunity to determine whether there is an association between training programs and the experience of physical or verbal aggression by staff in nursing homes and assisted living facilities using a survey that provides a nationally representative sample. Understanding whether training programs can mitigate occupational exposures to aggression may support interventions to reduce future workforce turnover [4].

The objective of this study was to leverage a newly released, nationally representative sample of the US paid dementia care workforce to determine the prevalence of physical and verbal aggression and whether there was an association between training programs and the frequency of physical or verbal aggression. We hypothesized that effective training would be associated with a reduction in the experience of physical and verbal aggression from residents. By identifying the association using a large, weighted, nationally representative dataset, we can inform policy, training standards, and workforce development to support the paid dementia care workforce in the United States.

## Methods

### Data

This study used wave 1 (2024) data from the nursing home and assisted living surveys collected by the National Dementia Workforce Study (NDWS) [10]. The NDWS is a multiyear, federally funded data infrastructure initiative designed to generate nationally representative data on the US dementia care workforce. The study conducted a 2-stage cluster sampling design of establishment- and individual-level participants from nursing homes and assisted living facilities to produce probability-based estimates of the national dementia care workforce population [11]. Survey weights were developed by NDWS statisticians and survey designers to permit nationally representative inference at the organization and staff levels, accounting for complex survey design, stratification, and clustering.

### Ethical Considerations

This study was approved by the University of Alabama at Birmingham Institutional Review Board (IRB-300015390). This analysis and reporting adhered to the Reporting of Studies Conducted Using Observational Routinely-Collected Data (RECORD) statement for the reporting of studies conducted using observational data.

### Dependent Variables

Our primary dependent variable was the presence or absence of physical or verbal aggression. To classify physical or verbal aggression, we used question 42 on the nursing home and assisted living surveys, which reads “In your job at this community over the past year, how often have you experienced the following?” We assessed the presence of physical aggression using subitem I, which reads “Hitting or other physical aggression from residents,” and verbal aggression using subitem J, which reads “Yelling or other verbal aggression from residents.” Potential responses were “never,” “rarely,” “sometimes,” and “often.” We dichotomized these variables as “not frequently” (encompassing “never” or “rarely”) and “frequently” (encompassing “sometimes” or “often”) because the original distribution did not meet the proportional odds assumptions of the Brant test, precluding analysis via linear or ordinal logistic regression.

Training was operationalized to reflect respondents’ perceived preparedness to manage aggressive behaviors in residents with dementia rather than the presence or absence of specific training modalities. Although the NDWS wave 1 surveys include items assessing exposure to formal and informal dementia-specific training (items NH4 and NH5 for nursing homes and AL4 and AL5 for assisted living facilities), these items ask broadly about training. Because the primary focus of this analysis was preparedness to handle aggressive behaviors, we used the global preparedness items (NH6 and AL6), which ask respondents whether their training prepared them to care for residents with dementia. These items capture respondents’ self-assessment of readiness rather than training format and were therefore better aligned with our analytic objective of examining perceived preparedness for challenging behavioral situations.

### Independent Variables

Independent variables included sex, age, race, ethnicity, feeling well-prepared by training to manage participant behaviors, rurality, and licensure. Licensure was determined by currently holding a registered nurse or licensed practical nurse license, whereas “nonlicensed” encompassed all other nonlicensed staff, including certified nursing assistants, home health aides, and medication assistants. We also included variables for directly working with residents and being a double-duty caregiver, where a staff member also provided care for an adult child at home.

Table 1 shows the variables and their item numbers and operationalizations.

**Table 1.** Variables, item numbers, and operationalizations.

Analytic variable	NDWS^a^ item number	Item wording (abbreviated)
Licensure	NH1 or AL1	Do you currently hold any professional license(s)?
Direct care	NH35 or AL35	Do you provide direct care to residents with Alzheimer’s disease or other dementias?
Educational level	NH3 or AL3	What is the highest level of education you have completed?
Sex	NH56 or AL56	What is your sex? (Male or female; masked in PUF^b^)
Age (mean, SD, and range)	NH48 or AL48	What year were you born? (Used to calculate age; masked in PUF)
Race	NH50 or AL50	What is your race? (Categories masked or collapsed in PUF)
Ethnicity	NH49 or AL49	Are you Hispanic, Latino/a, or of Spanish origin?
Family caregiver	NH37a-f or AL37a-f	Are you currently or have you ever been a family caregiver for a person with Alzheimer’s disease or other dementias? (Item series)
Physical aggression	NH38a-i or AL38a-i	How often have residents you care for done the following... (eg, hit, kicked, or physically hurt someone)?
Verbal aggression	NH38a-i or AL38a-i	How often have residents you care for yelled at, threatened, or verbally abused staff?
Training preparedness	NH6 or AL6	Overall, how well did your training prepare you to care for residents with Alzheimer’s disease or other dementias?
Rurality	Derived (facility level)	Rural vs nonrural classification derived from facility geographic indicators described in the NDWS wave 1 survey report (not a single staff-reported item)

a NDWS: National Dementia Workforce Study.

b PUF: Public Use File.

### Statistical Analysis

#### Overview

Descriptive statistics were used to summarize the characteristics of the included formal caregivers. Means and SDs were reported for continuous variables, whereas frequencies and percentages were reported for categorical variables. All descriptive estimates account for the complex survey design using the NDWS-provided sampling weights. All analyses were conducted in R (3.4.2; R Foundation for Statistical Computing). Missing data were handled via listwise deletion and were not imputed given the large, weighted sample size and the potential for weighted imputation to bias results.

#### Univariate Modeling

Chi-square and 2-tailed *t* tests were used to examine the association between the independent variables and a dichotomous high vs low frequency of experiencing physical or verbal aggression from residents.

#### Regression Modeling

Penalized logistic regression models using the Firth bias-reduced likelihood were fit to determine the strength and direction of the effect of feeling well-prepared by training programs on the dichotomous high vs low frequency of experiencing physical or verbal aggression from residents in nursing homes or assisted living facilities. NDWS-provided survey weights were used to represent the national dementia care workforce population. Clustering was accounted for in the survey object by specifying the primary sampling unit rather than a random effect included in the regression model as the cluster is a product of the NDWS sampling scheme rather than a multilevel structure of interest. Variance inflation factors were used to identify potentially problematic collinearity, with a variance inflation factor threshold of 10 or higher identified as problematic collinearity.

#### Supplementary Analysis

We compared respondents included in the regression models (complete cases) with the full survey sample for our variables of interest, incorporating survey weights. We also conducted an additional supplementary regression model that included ethnicity, despite ethnicity exhibiting potentially problematic collinearity, due to the established importance of ethnicity in the dementia care workforce [12,13].

## Results

### Overview

The dementia care workforce in nursing homes (n=896,320) was primarily female (828,558/896,320, 92.4%), White (440,158/896,320, 49.1%), and non-Hispanic (778,249/896,320, 86.8%), with a mean age of 41.49 (SD 12.65) years. Less than half (365,452/896,320, 40.8%) reported frequently experiencing physical aggression from residents, whereas more than half (470,536/896,320, 52.5%) reported frequently experiencing verbal aggression from residents. More than two-thirds (614,561/896,320, 68.6%) reported feeling that their training prepared them well to manage resident behaviors.

The dementia care workforce in assisted living facilities (n=738,856) was primarily female (631,070/738,856, 85.4%), White (490,181/738,856, 66.3%), and non-Hispanic (650,020/738,856, 88%), with a mean age of 41.36 (SD 13.94) years. More than one-quarter (198,964/738,856, 26.9%) reported frequently experiencing physical aggression from residents, whereas just under one-third (223,343/738,856, 30.2%) reported frequently experiencing verbal aggression from residents. Almost three-quarters (527,621/738,856, 71.4%) reported feeling that their training prepared them well to manage resident behaviors.

Table 2 shows the participant characteristics.

**Table 2.** Participant characteristics.

	Assisted living facilities (weighted n=738,856)	Nursing homes (weighted n=896,320)	Assisted living facilities (unweighted n=447)	Nursing homes (unweighted n=394)
Licensure, n (%)				
Licensed	74,140 (18.9)	295,052 (34.3)	60 (13.4)	126 (41.9)
Nonlicensed	318,778 (81.1)	563,909 (65.7)	387 (86.6)	233 (59.1)
Direct care, n (%)				
Direct care to residents	663,615 (89.9)	838,753 (93.6)	388 (88.8)	365 (92.6)
Not providing direct care	75,241 (10.2)	57,566 (6.4)	59 (11.2)	29 (7.4)
Educational level, n (%)				
Associate degree	76,224 (10.3)	96,090 (10.7)	45 (10.1)	47 (11.9)
Bachelor’s degree or higher	23,025 (3.1)	99,805 (11.1)	47 (10.5)	50 (12.7)
Lower than college	601,914 (81.5)	525,309 (58.6)	312 (70.0)	229 (58.1)
Vocational diploma or certificate	37,633 (5.1)	175,116 (19.5)	42 (9.4)	68 (17.3)
Sex, n (%)				
Female	631,070 (86.5)	828,558 (93.5)	388 (88.8)	353 (91.9)
Male	98,639 (13.5)	58,064 (6.5)	49 (11.2)	31 (8.1)
Age (y)				
Mean (SD)	41.36 (13.94)	41.49 (12.65)	40.596 (14.104)	40.222 (12.721)
Range	20.0-80.0	20.0-73.0	20-80	20-73
Race, n (%)				
Black or African American	126,423 (17.1)	367,459 (41)	83 (18.6)	104 (26.4)
Other	122,251 (16.5)	88,703 (9.9)	113 (25.3)	38 (9.6)
White	490,181 (66.3)	440,158 (49.1)	251 (56.2)	252 (64)
Ethnicity, n (%)				
Not Hispanic	650,020 (88.8)	778,249 (87.4)	356 (80)	351 (89.5)
Hispanic	82,350 (11.2)	112,085 (12.6)	89 (20)	41 (10.5)
Family caregiver, n (%)				
No	564,091 (76.4)	690,171 (77.3)	340 (76.2)	309 (78.6)
Yes	174,723 (23.6)	202,720 (22.7)	106 (23.8)	84 (21.4)
Physical aggression, n (%)				
Rarely or never	539,892 (73.1)	530,868 (59.2)	297 (66.4)	257 (65.2)
Sometimes or often	198,964 (26.9)	365,452 (40.8)	150 (33.6)	137 (34.8)
Verbal aggression, n (%)				
Rarely or never	515,513 (69.8)	425,784 (47.5)	265 (59.3)	201 (51)
Sometimes or often	223,343 (30.2)	470,536 (52.5)	182 (40.7)	193 (49)
Training preparedness, n (%)				
Not well-prepared	211,235 (28.6)	281,758 (31.4)	131 (29.3)	121 (30.7)
Well-prepared	527,621 (71.4)	614,561 (68.6)	316 (70.7)	273 (69.3)
Rurality, n (%)				
Not rural	484,502 (65.6)	675,002 (75.3)	429 (96)	313 (79.4)
Rural	254,354 (34.4)	221,318 (24.7)	18 (4)	81 (20.6)

### Univariate Modeling

In univariate modeling in nursing homes, race (*P*=.03) and training preparedness (*P*=.002) were associated with experiencing physical aggression. Family caregiver status (*P*=.04) and training preparedness (*P*=.01) were associated with experiencing verbal aggression.

In univariate modeling in assisted living facilities, race (*P*=.002) and training preparedness (*P*<.001) were associated with experiencing physical aggression. Sex (*P*=.02), race (*P*=.002), and training preparedness (*P*<.001) were associated with experiencing verbal aggression.

Table 3 shows the univariate modeling results.

**Table 3.** Univariate modeling results.

	Assisted living facilities, *P* value		Nursing homes, *P* value	
	Physical aggression	Verbal aggression	Physical aggression	Verbal aggression
Age (mean and SD)	.49	.36	.18	.35
Licensure	.29	.31	.21	.30
Direct care role	.01	.01	.67	.35
Sex	.05	.02	.49	.27
Race	.002	.002	.03	.14
Ethnicity	.08	.33	.23	.36
Family caregiver	.38	.31	.30	.04
Training preparedness	<.001	<.001	.002	.01
Rural	.79	.50	.33	.82

### Regression Modeling

In the fully adjusted model estimating the likelihood of reporting frequent physical aggression in nursing homes, feeling well-prepared by training for managing resident behaviors (odds ratio [OR] 0.40, 95% CI 0.223-0.711; *P*=.01) was associated with a lower likelihood of reporting frequent physical aggression.

In the fully adjusted model estimating the likelihood of reporting frequent verbal aggression in nursing homes, feeling well-prepared by training for managing resident behaviors (OR 0.50, 95% CI 0.299-0.844; *P*=.02) was associated with a lower likelihood of reporting frequent verbal aggression.

In the fully adjusted model estimating the likelihood of reporting physical aggression in assisted living facilities, feeling well-prepared by training for managing resident behaviors (OR 0.14, 95% CI 0.036-0.556; *P*=.01) and being a family caregiver (OR 0.19, 95% CI 0.043-0.778; *P*=.03) were associated with a lower likelihood of reporting frequent physical aggression.

In the fully adjusted model estimating the likelihood of reporting verbal aggression in assisted living facilities, working in a direct care role (OR 5.09, 95% CI 1.223-21.194; *P=*.03) was associated with a higher likelihood of reporting frequent verbal aggression, and feeling well-prepared by training for managing resident behaviors (OR 0.07, 95% CI 0.014-0.354; *P*<.001) and being a family caregiver (OR 0.18, 95% CI 0.061-0.516; *P*<.001) were associated with a lower likelihood of verbal aggression.

Table 4 shows the regression modeling results.

**Table 4.** Regression modeling results.

Term	Assisted living facilities				Nursing homes			
	Physical aggression		Verbal aggression		Physical aggression		Verbal aggression	
	Odds ratio (95% CI)	*P* value	Odds ratio (95% CI)	*P* value	Odds ratio (95% CI)	*P* value	Odds ratio (95% CI)	*P* value
Intercept	0.52 (0.066-4.052)	.54	1.31 (0.153-11.254)	.81	3.99 (0.973-16.384)	.07	3.50 (1.041-11.776)	.06
Licensed	0.34 (0.067-1.72)	.20	0.33 (0.044-2.425)	.29	1.32 (0.612-2.864)	.48	1.18 (0.605-2.286)	.64
Direct care role	4.86 (0.761-31.079)	.11	5.09 (1.223-21.194)	.03	0.97 (0.282-3.365)	.97	1.22 (0.287-5.207)	.79
Male sex	2.79 (0.8-9.723)	.12	2.68 (0.872-8.24)	.10	0.57 (0.182-1.77)	.34	0.45 (0.166-1.122)	.13
Age	0.99 (0.954-1.034)	.76	0.99 (0.938-1.035)	.56	0.98 (0.951-1.005)	.13	0.98 (0.956-1.01)	.23
Non-White race	1.85 (0.452-7.582)	.40	2.54 (0.416-15.378)	.32	0.63 (0.345-1.142)	.14	0.62 (0.37-1.042)	.09
Family caregiver	0.19 (0.043-0.778)	.03	0.18 (0.061-0.516)	<.001	1.52 (0.857-2.709)	.17	2.33 (0.857-6.315)	.11
Training preparedness (well-prepared)	0.14 (0.036-0.556)	.01	0.07 (0.014-0.354)	<.001	0.40 (0.223-0.711)	.01	0.50 (0.299-0.844)	.02
Rurality	1.24 (0.264-5.817)	.79	0.89 (0.176-4.546)	.89	0.84 (0.317-2.206)	.72	1.13 (0.763-1.659)	.56

### Supplementary Modeling

#### Sensitivity Analysis

In the sensitivity analysis, we did not find statistically substantial differences between the complete-case sample included in the regression modeling and those who were missing data for key demographic variables (Table 5).

**Table 5.** Sensitivity analysis comparing complete cases.

Variable	Nursing homes, *P* value	Assisted living facilities, *P* value
Age	.14	.64
Sex	.23	.13
Race	.62	.23
Training preparedness	.55	.91
Ethnicity	.24	.32

#### Regression Modeling

In the supplementary analysis of assisted living facilities, feeling that training prepared them well for managing resident behaviors (OR 0.13, 95% CI 0.04-0.56; *P*=.01) and family caregiver status (OR 0.20, 95% CI 0.05-0.75; *P*=.03) were associated with a lower likelihood of reporting frequent physical aggression. Hispanic ethnicity (OR 14.03, 95% CI 2.77-71.01; *P*<.001) was associated with a higher likelihood of reporting frequent physical aggression.

In the supplementary analysis of assisted living facilities, a direct care role (OR 4.74, 95% CI 1.21-18.68; *P*=.04) was associated with a higher likelihood of reporting frequent verbal aggression. Family caregiver status (OR 0.18, 95% CI 0.06-0.51; *P*<.001) and feeling prepared by training (OR 0.07, 95% CI 0.01-0.37; *P*=.01) were associated with a lower likelihood of reporting frequent verbal aggression.

In the supplementary analysis of nursing homes, feeling prepared by training (OR 0.48, 95% CI 0.26-0.89; *P*=.03) was associated with a lower likelihood of reporting frequent verbal aggression.

Table 6 shows the supplementary regression modeling results.

**Table 6.** Supplementary regression modeling results.

Term	Assisted living facilities				Nursing homes			
	Physical aggression		Verbal aggression		Physical aggression		Verbal aggression	
	Odds ratio (95% CI)	*P* value	Odds ratio (95% CI)	*P* value	Odds ratio (95% CI)	*P* value	Odds ratio (95% CI)	*P* value
Intercept	0.05 (0.00‐0.48)	.02	0.58 (0.04‐8.78)	.70	2.05 (0.60‐7.02)	.27	2.14 (0.58‐7.95)	.27
Licensed	0.27 (0.04‐1.76)	.19	0.3 (0.03‐2.69)	.30	1.39 (0.57‐3.38)	.48	1.2 (0.59‐2.44)	.62
Direct care role	4.25 (0.90‐20.12)	.08	4.74 (1.21‐18.68)	.04	1.06 (0.33‐3.34)	.93	1.3 (0.33‐5.14)	.71
Male sex	2.44 (0.67‐8.84)	.19	2.51 (0.81‐7.79)	.12	0.54 (0.16‐1.75)	.31	0.43 (0.16‐1.16)	.12
Age	1 (0.96‐1.04)	.91	0.99 (0.94‐1.04)	.65	0.98 (0.95‐1.00)	.10	0.98 (0.96‐1.01)	.21
Non-White race	2.23 (0.52‐9.51)	.29	2.84 (0.44‐18.32)	.28	0.65 (0.32‐1.30)	.24	0.63 (0.36‐1.13)	.14
Hispanic ethnicity	14.03 (2.77‐71.01)	<.001	2.4 (0.47‐12.25)	.30	2.18 (0.52‐9.12)	.30	1.74 (0.62‐4.85)	.31
Family caregiver	0.20 (0.05‐0.75)	.03	0.18 (0.06‐0.51)	<.001	1.48 (0.86‐2.56)	.18	2.26 (0.85‐6.00)	.12
Training preparedness (well-prepared)	0.13 (0.04‐0.56)	.01	0.07 (0.01‐0.37)	.01	0.38 (0.18‐0.78)	.17	0.48 (0.26‐0.89)	*.03*
Rurality	0.98 (0.21‐4.46)	.98	0.81 (0.15‐4.36)	.81	0.83 (0.32‐2.18)	.71	1.14 (0.78‐1.65)	.51

## Discussion

### Overview of Findings

In both nursing homes and assisted living facilities, training programs for handling resident behaviors were associated with a lower likelihood of experiencing physical aggression, and in assisted living facilities, these training programs were associated with a lower likelihood of experiencing verbal aggression.

### Comparison With Prior Literature

Prior work emphasizes the importance of dementia-specific training to manage resident behaviors. Livingston et al [14] demonstrated in a systematic review of 33 studies that aggression can be mitigated through caregiver activities, especially person-centered approaches to care that systematically support resident behaviors. Other reviews have found that aggression prevention in residential facilities requires staff to be equipped with knowledge and skills to intervene early to prevent situational escalation into physically or verbally aggressive behaviors, although Seitz et al [15] concluded that large-scale studies of staff training in nursing homes and residential facilities should examine whether these results are robust beyond protocol-driven interventions. Our results indicate that, on a nationally representative scale, there is an association between a reduced frequency of aggressive behaviors and perception that trainings prepared staff to manage participant behaviors. While the cross-sectional nature of the NDWS precludes causal speculation, other work has hypothesized that training that includes early recognition and intervention for problematic behaviors can reduce aggression in residents with dementia [16,17].

Our finding that being a double-duty caregiver, or caring for a person at home, was associated with a reduced likelihood of experiencing aggressive behaviors in assisted living facilities was initially surprising. However, this correlation could be due to their experience in understanding and recognizing escalating cascades of behaviors when caring for another person in the home [18]. Limited literature exists to determine the specific skills that double-duty caregivers apply in both home and long-term care settings, although a future study could determine the influence of home-based caregiving on long-term care strategies to manage aggression.

### Limitations

The large, well-conducted survey methodology and robust weighting procedures provide strength to our findings. However, care is needed in the interpretation of results due to the cross-sectional nature of the study; causality cannot be assumed. Our findings indicate that perceived training efficacy for managing resident behaviors was linked to a decrease in the frequency of aggressive incidents; however, the specific elements of the training contributing to this effect remain undetermined. On the other hand, staff experiencing lower aggression may create the perception that their training sufficiently prepared them to manage resident behaviors. Future interventional work should determine the causal path. Additionally, the study power was limited by the initial sampling frame rather than the weighted sample. There could have been aspects of the study sample that biased the results, although the robust weighting procedures and clustering in the survey design should mitigate this effect. We treated training preparedness as dichotomous due to small cell sizes in the “not at all prepared” group. While this speaks to the level of training received at nursing homes and assisted living facilities, future work should determine whether there is a stepwise change between similar categories as there could be a meaningful difference between feeling not at all prepared and somewhat prepared.

### Conclusions

In a nationally representative sample of the dementia care workforce, effective training programs were associated with worker-reported reduced experiences of physical and verbal aggression in both nursing homes and assisted living facilities. The specific components of training programs that may support effectiveness remain unknown.

## Abbreviations

NDWS: National Dementia Workforce Study
OR: odds ratio
RECORD: Reporting of Studies Conducted Using Observational Routinely-Collected Data
